# Copolymer of VDF/TFE as a Promising Polymer Additive for CsH_2_PO_4_-Based Composite Electrolytes

**DOI:** 10.3390/membranes13050520

**Published:** 2023-05-17

**Authors:** Yuri Kungurtsev, Irina Bagryantseva, Valentina Ponomareva

**Affiliations:** 1 Institute of Solid State Chemistry and Mechanochemistry SB RAS, 630090 Novosibirsk, Russia; 2 Department of Natural Sciences, Novosibirsk State University, 630090 Novosibirsk, Russia

**Keywords:** cesium dihydrogen phosphate, proton membranes, polymer electrolyte, proton conductivity, thin films, fluoropolymers, copolymers

## Abstract

The composite polymer electrolytes (1-x)CsH_2_PO_4_-xF-2M (x = 0–0.3) have been first synthesized and their electrotransport, structural, and mechanical properties were investigated in detail by impedance, FTIR spectroscopy, electron microscopy, and X-ray diffraction methods. The structure of CsH_2_PO_4_ (P2_1_/m) with salt dispersion is retained in the polymer electrolytes. The FTIR and PXRD data are consistent, showing no chemical interaction between the components in the polymer systems, but the salt dispersion is due to a weak interface interaction. The close to uniform distribution of the particles and their agglomerates is observed. The obtained polymer composites are suitable for making thin highly conductive films (60–100 μm) with high mechanical strength. The proton conductivity of the polymer membranes up to x = 0.05–0.1 is close to the pure salt. The further polymers addition up to x = 0.25 results in a significant decrease in the superproton conductivity due to the percolation effect. Despite a decrease, the conductivity values at 180–250 °C remain high enough to enable the use of (1-x)CsH_2_PO_4_-xF-2M as a proton membrane in the intermediate temperature range.

## 1. Introduction

Solid Acid Fuel Cell (SAFC) is a promising chemical source of electrical power. Materials based on acid salts of alkali metals with the general formula M_n_H_m_(AO_4_)_p_ (M = Cs, Rb, NH_4_^+^, A = P, As, S, Se, where m, n, p are integers) have high proton conductivity (~10^−3^–10^−2^ S/cm), which is due to the presence of superionic phase transition with restructuring of hydrogen bond network and its disordering. These materials are widely used as proton-conducting membranes for medium-temperature fuel cells [1,2,3,4,5]. However, the most developed are low-temperature fuel cells with Nafion-type proton-exchange membranes [6,7,8], low operating temperatures (≤100 °C) result in a number of disadvantages such as the complicated process of water management and the sensitivity of electrodes to fuel impurities. Medium-temperature operating range (130–350 °C) of SAFC is optimal in terms of energy consumption and the possibility of using inexpensive structural materials. The higher rate of electrode reactions allows the use of less pure fuels with the impurities concentrations reaching values of 10% of CO and 100 ppm of H_2_S [3,9]. The mechanism of the proton transport of acid salts is implemented in the solid state and does not require a high degree of membrane moisture. In addition, the solid nature of the electrolyte makes the acid salt membrane more resistant to fuel and gas crossover compared to a hydrated Nafion membrane.

Active research of the proton conductors has been conducted since the mid-twentieth century. CsH_2_PO_4_ and CsHSO_4_ have the highest conductivity values [10,11]. These compounds are used as the solid electrolytes in the proton-conducting SAFC membranes. CsHSO_4_ can be reduced in a humidified hydrogen atmosphere with the formation of hydrogen sulfide, which can poison Pt-electrode [12,13]. Therefore, CsH_2_PO_4_ (CDP) is most often used as a solid electrolyte for membrane-electrode assembly (MEA). CDP exhibits the high proton conductivity values of 6·10^−2^ S·cm^−1^ at 230 °C in the superionic state. In the monoclinic phase (P2_1_/m), CDP has conductivity values of about 10^−7^ S·cm^−1^ and a phase transition occurs from the non-conductive monoclinic phase to the superionic cubic phase (Pm-3m) at 230 °C [14,15]. The mechanism of proton transfer in the superionic phase is due to the phosphate tetrahedra reorientation with the consequent proton hopping. SAFCs with a cesium dihydrogen phosphate electrolyte were first reported in 2004 by Haile [1]. Such fuel cells achieve high values of a peak power density of ~415 mW/cm^2^ at T = 250 °C [5]. However, some improvements in the cathodic processes acting as a current-limiting factor in the fuel cells based on CsH_2_PO_4_ are necessary [16,17]. It is required to achieve greater contact between the substances at the three-phase boundary. In most cases, the decrease in the electrochemical characteristics of the fuel cells was caused by the imperfections in the electrodes and their compositions, their interfacial surfaces, the method of application, and their stability over time [18]. However, CDP can form some meta- and pyrophosphates at high temperatures, which are non-conductive phases [19,20] and are drawbacks as a membrane material. Cesium dihydrogen phosphate decomposes at temperatures above 230 °C to form:

CsH_2_PO_4_ → CsPO_3_ + H_2_O

CDP is soluble in water, which results in some difficulties for fuel cell performance. Additionally, the salt is brittle, which negatively affects the performance of SAFCs. The phase transition of CDP, in which there is a jump in the conductivity by four orders of magnitude, is also an undesirable phenomenon. Partially, these disadvantages can be eliminated by adding various compounds to cesium dihydrogen phosphate. Currently, there is intensive research into suitable thermally stable polymer compositions and methods of synthesizing organo-inorganic membranes. The ability to modify the properties of CsH_2_PO_4_ and synthesize the proton-conducting electrolytes with high mechanical strength and high conductivity at medium temperatures (up to the phase transition) is not only of scientific interest but also of technological interest since CsH_2_PO_4_ is a potential membrane material for medium-temperature fuel cells. Two methods of doping CDP are widely used—homogeneous and heterogeneous doping of the acid salts. In homogeneous doping, different cations or anions are incorporated into the crystalline structure to form the solid solutions. The work in this direction is actively being carried out, as reported [21,22,23,24,25]. In heterogeneous doping, a non-conducting additive is added to the acid salt, usually to improve the mechanical properties of cesium dihydrogen phosphate, and to distribute the salt particles in the matrix of the heterogeneous additive. The earlier works [26,27,28,29] reported on producing CDP-polymer additive systems. It has been shown that adding, for example, epoxy resin significantly improves the mechanical properties of the obtained membranes [27]. According to the literature, membranes made of polymer composites show stable performance in fuel cell testing.

Using fluoropolymers as heterogeneous additives for CDP is also possible. Fluorine-containing polymers are thermally stable, chemically inert, hydrophobic, excellent insulators, and mechanically strong. Fluoropolymers have been widely utilized in the field of power engineering such as the separators in lithium-ion batteries and supercapacitors [30,31,32]. Some of them are soluble in dimethylformamide (DMF) or ketones, which allow the production of films based on them, potentially reducing ohmic losses. Fluoropolymers have high tensile strength values (up to 80 MPa), confirming their mechanical strength. The properties of fluoropolymers make their use as dopants for cesium dihydrogen phosphate promising. Earlier the reports were made of “CDP—fluoropolymer” systems, where such polymers as ultrafine polytetrafluoroethylene (UPTFE), polyvinylidene fluoride (PVDF), VDF copolymers with hexafluoropropylene (SKF-26), with tetrafluoroethylene (F-42), were used as additives [26,28,33,34]. Specifically, fluoroplastic-2M, with a commercial name F-2M, can be used as it consists of modified PVDF composition. Its composition can be represented as follows [-CH_2_-CF_2_-]_n_·[-CF_2_-CF_2_-]_m_ where m/n = 0.05. Unlike pure PVDF, F-2M is more elastic, it has the highest strength, hardness (up to 90 MPa on the Brinell scale), and good piezoelectric properties, and starts melting at 177 °C while decomposing at temperatures above 380 °C. F-2M has a reasonably high degree of crystallinity compared to the amorphous polymers. 

**Figure 1:**
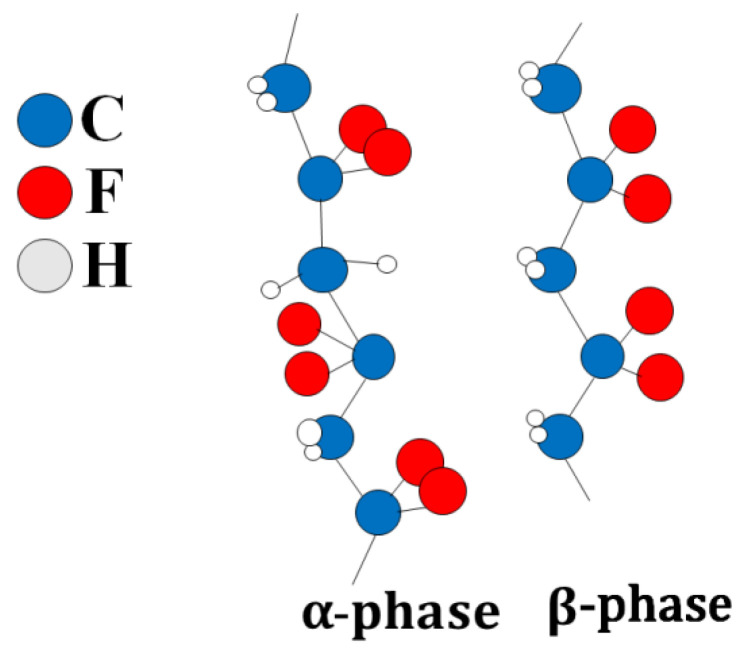
Alpha and beta phases of F-2M.Thus, F-2M has good mechanical properties and is dielectric with high chemical and thermal stability. The fact that thin films can be made from fluoroplastic-2M makes it a promising matrix for CDP. This article is devoted to the synthesis of the composite polymer electrolytes (1-x)CsH_2_PO_4_-xF-2M in the range of compositions (x = 0–0.3) (x is the weight fraction of F-2M) and the investigation of electrotransport, structural, and mechanical properties. The development of polymer systems of this type is of considerable interest due to the chemical, thermal, and mechanical characteristics of fluoroplastic-2M and permits to create the thin highly conductive proton membranes, mechanically strong and gas-tight for medium-temperature electrochemical devices.

PVDF has several polymorphic states: α, β, γ, δ-phases. It is characterized by a regular structure in which most of the PVDF units are connected head-to-tail, and only a small number are linked head-to-head [35,36] (Figure 1). The C-F bonds are polar, and the maximum dipole moment is achieved when all dipoles of the polymer molecule are aligned in the same direction. This structure corresponds to the β-phase of PVDF [37]. Optimal piezoelectric characteristics of the polymer are achieved when it contains the β-phase. Alpha-crystallite dipole moments are oriented in the opposite direction, resulting in zero polarization.

## 2. Materials and Methods

To obtain CsH_2_PO_4_, the precipitation was carried out from an aqueous solution containing Cs_2_CO_3_ and H_3_PO_4_ in a stoichiometric ratio using isopropanol as a solvent:

Cs_2_CO_3_ + 2H_3_PO_4_→ 2CsH_2_PO_4_ + CO_2_ + 3H_2_O

The samples for measuring the conductivity with a composition of x = 0.05–0.15 were pressed with fine-dispersed silver electrodes. The tablets for measuring the conductivity were made by mechanically grinding a mixture of F-2M and the salt in an agate mortar, followed by uniaxial pressing at 300 MPa into the dense tablets (diameter 6 mm, thickness 2–3 mm, relative density 96–98%) with thin silver electrodes. The samples with a composition of x = 0.2–0.25 were obtained by casting the thin films with a thickness of up to 100 μm. The films were obtained from a suspension of the salt and the polymer in DMF and acetone (1:1, vol.), which were then applied to the substrate using an applicator with a predetermined thickness (350 μm). After obtaining the film, it was prepressed to densify the membranes, and then again prepressed with fine-dispersed silver.

The proton conductivity of the (1-x)CsH_2_PO_4_-xF-2M membranes was measured under humid conditions using argon passed through a bubbler with water, which was heated to 70 °C (p_H2O_ = 0.3 atm., gas flow rate 50 mL/min.) to prevent CDP decomposition. The impedance measurements were performed for the samples in the form of films (l < 100 μm, x = 0.2 and 0.25) or tablets with two silver electrodes. The samples were heated and cooled several times in the temperature range from 25 to 250 °C at a rate of 1–2 °C/min to obtain the temperature dependencies of the (1-x)CsH_2_PO_4_-xF-2M conductivity. The measurements were carried out under the conditions of humidified flowing air, argon (P_H2O_ ~ 0.3 atm.) with a flow rate of 50 mL/min. The gas flow rate was fixed using a regulator (Modern laboratory equipment, Novosibirsk, Russia). The proton conductivity of the samples was measured in a two-electrode cell in the cooling regime (~1 degree/min) using the impedance spectroscopy on the E7-20 (MNIPI, Minsk, Belarus) and the Instek (Daejeon, Republic of Korea) impedance analyzers in the frequency range of 0.1 Hz–1 MHz and 12 Hz–200 kHz, respectively. The conductivity of membranes was calculated using the following formula:

$$\sigma =\frac{l}{Z*S}$$

*Z*—resistance values for the sample, *l—*sample thickness, *S*—sample surface area. Scanning electron microscope Hitachi TM 1000 was used to evaluate the size and shape of particles. PXRD patterns were obtained on a Bruker D8 Advance diffractometer (λ_CuKα1_ = 1.5406 Å) with a one-dimensional Lynx-Eye detector and a K_β_ filter at room temperature. Fourier-transform infrared spectroscopy (FTIR) was used to investigate the structural features of the compounds, the nearest atom environment, and the hydrogen bonding network. The spectra in the attenuated total reflectance (ATR) mode were recorded on a Bruker Tensor 27 spectrometer. 

The tensile strength measurements were made on an Instron 5944 at a breaking of the thin-film membranes on the load. The tensile strength was referred to as the initial cross-sectional area of the sample. The samples with a double-bladed form with a working area (5 mm·20 mm) were stretched at a constant rate (5 mm/min) and the elongation was recorded.

## 3. Results and Discussion

The selection of the solvents is an important part of obtaining materials for the proton-conducting membranes. Using a solvent with a low boiling point, such as acetone, leads to the formation of pores in a thin film, so it is desirable to select a solvent so that the resulting film is gas-tight. DMF (boiling point = 151 °C) is a suitable solvent, but the film dries very slowly. However, the prolonged drying time may lead to the process of CDP recrystallization, which is an extremely undesirable process. Adding acetone to DMF can accelerate the drying time of the film. The membrane synthesis procedure by the tape casting method can also be improved by searching for the most optimal solvents, suspension application rate, gap height, drying mode, etc.

**Figure 2:**
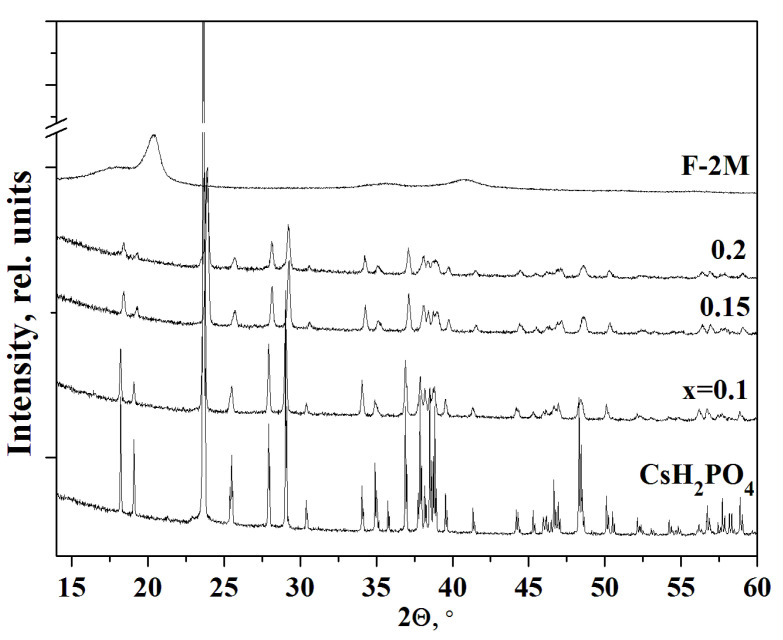
PXRD of polymer electrolytes (1-x)CsH_2_PO_4_-xF-2M of different compositions in comparison with pure CsH_2_PO_4_ and F-2M.

The PXRD pattern of CsH_2_PO_4_ (P2_1_/m) at room temperature completely coincided with [38] (Figure 2). F-2M substance has a sufficiently high degree of crystallinity for a polymer. According to the PXRD data, the monoclinic P2_1_/m phase of cesium dihydrogen phosphate is preserved for all the compositions of (1-x)CsH_2_PO_4_-xF-2M membranes obtained by different synthesis methods. No chemical interaction between the salt and the polymer was observed. With an increase of x, the intensities of the reflections corresponding to the pure salt decrease more significantly than the salt content in the polymer systems. With an increase in the weight fraction of the polymer up to x = 0.1–0.2, a further decrease in the intensity of the reflections and their broadening were observed. The peaks become broadened due to the salt dispersion. It is seen, the intensity of the peak is more than twice smaller for x = 0.1, while the salt content in the composite is 90%. The decreasing intensity of the CsH_2_PO_4_ reflections in the polymer composites and their broadening was observed with increasing F-2M fraction due to the significant dispersion of the salt particles. Since the structure of the salt remains unchanged in the polymer systems, its dispersion and a slight decrease in the unit cell parameters are the result of the interface interaction with the polymer matrix. Besides, the reflections in the polymer systems move towards the larger angles due to a slight decrease of the unit cell parameters of CsH_2_PO_4_ surrounded by the polymer matrix. Pure F-2M has reflections at 2θ ~ 20° and 41° corresponding to the β-phase of PVDF. The reflections of the polymer matrix do not appear on the diffraction patterns due to its low content up to x = 0.2. Thus, PXRD patterns show that the structure of CsH_2_PO_4_ (P2_1_/m) with considerable salt dispersion was retained in the studied polymer electrolytes.

**Figure 3:**
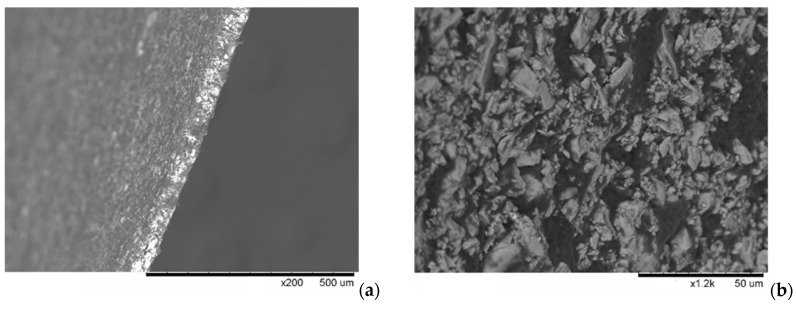
SEM images of the cross-section of the thin-film membrane with x = 0.3 (**a**) and x = 0.25 (**b**).

The SEM images were obtained for the (1-x)CsH_2_PO_4_-xF-2M, x = 0.25–0.3 samples (Figure 3). While the average particle size of the pure salt does not exceed 1–5 μm, the distribution of the salt in the polymer matrix can be considered rather uniform (Figure 3b). The method used is shown to be suitable for making the thin films up to 60–100 μm. The cross-section of the thin-film membrane does not exceed ~50–60 μm (Figure 3a). The obtained thin films of the proton membranes (x = 0.2–0.3) were non-porous. The conductivity of the obtained composite electrolytes and the distribution of the salt particles can be further improved by the pretreatment of the suspension of the salt particles in the polymer solution using a bead mill.

**Figure 4:**
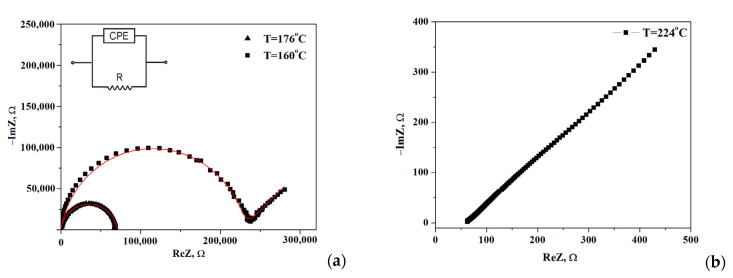
Impedance spectra of (1-x)CsH_2_PO_4_-xF2M (x = 0.2) at different temperatures 160, 176, (**a**) and 224 °C (**b**) (sample thickness l = 0.17 cm, surface area S = 0.3 cm^2^).

The impedance spectra of the composite polymer electrolytes (1-x)CsH_2_PO_4_-xF-2M are presented in Figure 4 for x = 0.2. Note, the impedance measurements were carried out for each point on the temperature dependence of the conductivity. Moreover, for all samples, the Nyquist plots (Z′ vs. Z″ as a parametric function of frequency) were repeatedly reproduced including 2–3 samples for each composition. The Nyquist plots at temperatures 176 °C and 224 °C show the dependencies for the high- and low-temperature regions, respectively. The impedance hodographs are represented by a semicircle with a much smaller radius at higher temperatures (T = 176 °C) and a semicircle combined with a straight line with a 45° angle to the abscissa at T = 160 °C. The resistance of the obtained electrolytes was estimated from the point of the intersection with the minimum of the imaginary component on the real axis. Impedance hodograph at T = 176 °C can be approximated by the equivalent circuit, consisting of a parallel combination of resistance^®^ and a constant phase element (CPE) (Figure 4a, inset) with the fitting parameters R = 7.06 × 10^4^ Ω, Q = 3.04 × 10^−10^ F, α = 0.92, where Q is the capacitance of the constant phase element, α is the degree of deviation from the values for pure capacitance. For the hodograph at a T = 160 °C semicircle is combined with the Warburg impedance and the fitting parameters R = 2.28 × 10^5^ Ω, Q = 5.75 × 10^−10^ F, α = 0.91, A_w_ = 1.44 × 10^5^. The approximation error was less than ≤2%. The impedance spectrum at high temperatures (T = 224 °C, Figure 4b) is represented by a straight line which is due to the fast proton conductivity through the electrolyte and corresponded to the electrode resistance at the lower frequencies. Such spectra are characteristic of superionic phases [3].

**Figure 5:**
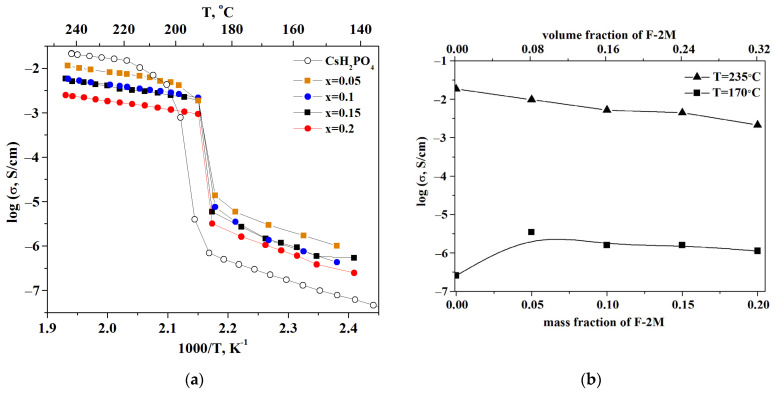
Temperature dependencies of proton conductivity (**a**) and isotherms (**b**) of (1-x)CsH_2_PO_4_-xF2M polymer electrolytes and initial CsH_2_PO_4_.

The observed jump in the proton conductivity of CsH_2_PO_4_ due to the superproton phase transition is four orders of magnitude (Figure 5a). The dependence of the proton conductivity on the temperature was investigated for the samples with different ratios of the polymer additives. For clarity, the temperature dependencies are shown in Arrhenius coordinates. The measurements were carried out for 2–3 samples for each composition. The temperature dependence of the proton conductivity in the composite polymer systems (1-x)CsH_2_PO_4_-xF-2M is similar to that of the original salt and is characterized by the low-temperature (LT) and high-temperature (HT) regions. The obtained tablets with x = 0.05; 0.1; 0.15 have approximately close values of the proton conductivity in the HT phase and decrease by up to five times compared to the CDP. The conductivity of the samples with x = 0.2 and x = 0.25, which were obtained in the form of the thin films, decreases by 1–1.5 orders of magnitude compared to the pure CsH_2_PO_4_. The activation energy of the conductivity for the polymer systems is equal to 0.4 eV for the superionic region and about 0.8 eV for the low-temperature range and is close to the pure CsH_2_PO_4_. Isotherms of the proton conductivity of (1-x)CsH_2_PO_4_-xF-2M composites for different temperatures depending on the mass and volume fraction of the polymer are presented in Figure 5b. In the low-temperature region (T = 170 °C), the proton conductivity of the polymer electrolytes increases with x growth due to the slight disordering and amorphization of acid salt. That is consistent with the XRD and FTIR data for CsH_2_PO_4_-F-2M composites.

In some cases, the conductivity of weakly conducting systems may be increased by several orders of magnitude compared to the pure salts by addition of the high dispersed additives, which can be classified as a composite effect [39,40,41]. However, the (1-x)CsH_2_PO_4_-xF-2M materials under consideration are representatives mainly of the “conductor-insulator” type systems. Accordingly, the isotherm in high-temperature region (T = 235 °C) linear decrease of proton conductivity due to «conductor-insulator» percolation effect is observed (Figure 5b). The proton conductivity of the systems starts to decrease already at a sufficiently small volumetric fraction of the polymer due to the dielectric nature of the polymer. A decrease in the proton conductivity was also observed for the similar systems “CsH_2_PO_4_-polymer” [26,27,29,33]. The proton conductivity of (1-x)CsH_2_PO_4_-xF-2M systems is close to the existing literature data for a number of the polymer systems [26]. Note that the samples obtained exhibit a wider temperature range for the superionic phase existence than the pure CDP. The conductivity jump due to the phase transition to the monoclinic phase shifts to the lower temperature range of more than 20 °C in the polymer (1-x)CsH_2_PO_4_-xF-2M systems (Figure 5a). These polymer systems differ markedly from the other polymer membranes based on CDP and create a perspective for decreasing the working temperature range of SAFCs. Despite the decrease in the proton conductivity, the hybrid inorganic-organic membranes have sufficiently high conductivity for use in medium-temperature fuel cells. The storage of sample x = 0.2 at high temperatures (T = 245 °C, pH_2_O ~ 0.3 atm) for ~4 h shows the stability of the proton conductivity values for the membranes (Figure S1).

**Figure 6:**
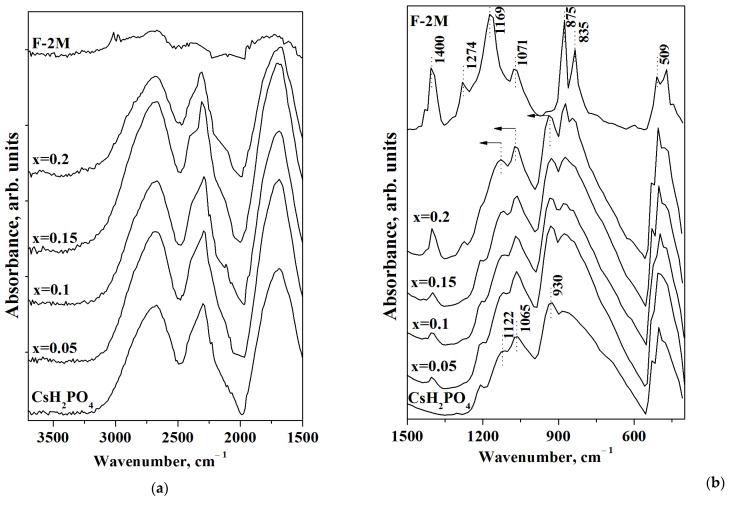
FTIR spectra in the region of the hydrogen bonds (**a**) and PO_4_ tetrahedra vibrations (**b**) of (1-x)CsH_2_PO_4_-xF-2M for different compositions in a comparison with CsH_2_PO_4_ and F-2M (arrows show the direction of the a.b. displacement).

FTIR spectra of CsH_2_PO_4_, F-2M, and (1-x)CsH_2_PO_4_-xF-2M polymer composites are presented in Figure 6. The FTIR spectrum of CsH_2_PO_4_ has two ranges: the intensive absorption bands (a.b.) at 2800–1700 cm^−1^ related to the stretching (ν_OH_) and the overtones of the bending vibrations of OH^−^ groups involved in hydrogen bonds (Figure 6a), and 1300–500 cm^−1^, corresponding mainly to the spectral range of νPO_4_ tetrahedra (Figure 6b). Figure 6a shows the system of the strong hydrogen bonds for CsH_2_PO_4_ [42]. The range of 1500–500 cm^−1^ of FTIR spectra for F-2M corresponds to the main characteristic absorption bands. The intensive a.b. 1400 and 1169 cm^−1^ are related to the antisymmetric and symmetric stretching vibrations of the CF_2_ groups, respectively, while the a.b. at 1071 cm^−1^ is associated with the antisymmetric stretching vibrations of fluorine in CF_2_ [43,44]. The intensive absorption bands of 1274 and 875 cm^−1^ deal with CH_2_ wagging and stretching vibrations, while the a.b. 835 cm^−1^ belong to the rocking CH_2_ and stretching CF2 groups. The absorption band 509 cm^−1^ corresponds to the CF_2_ bonds of the carbon chain [45,46]. All the absorption bands of CsH_2_PO_4_ remain in the polymer systems with an increase in the weight fraction of the F-2M matrix, and FTIR spectra of the polymer membranes are similar to the pure CsH_2_PO_4_. Further, with the increasing polymer fraction, the additional absorption bands in the spectral range of PO_4_ tetrahedra appear due to the superposition of the a.b. of the components. With x increase up to x = 0.2 the a.b. in the range of 1400, 1274, 875, 835, and 509 cm^−1^ appear in the polymer membranes in the addition to FTIR spectrum of CsH_2_PO_4_. It should be noted that there is only a slight change in the positions of absorption bands of CsH_2_PO_4_ in the polymer composites with the increase of F-2M. The absorption bands of 930 and 1122 cm^−1^ shift to the 939 and 1133 cm^−1^, respectively at x = 0.2, which is related to the strengthening of P-O bonds in the polymer system. Thus, the data from both FTIR spectra and XRD are consistent and confirm the stability of the CsH_2_PO_4_ in the polymer composites with insignificant changes due to the interface interaction of the components and the salt dispersion. It is likely that the weak interface interaction with the negligible strengthening of the P-O bonds determines the shift in the phase transition to the lower temperatures and the expansion of the range of the high-temperature phase of CsH_2_PO_4_ in a polymer matrix. 

**Figure 7:**
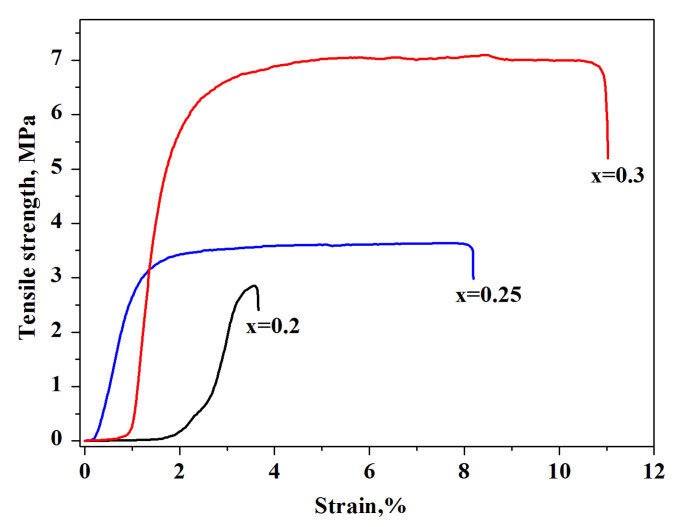
Stress–strain curve of the (1-x)CsH_2_PO_4_-xF-2M membranes of different compositions.

For the thin-film membranes (x = 0.2–0.3 and thickness of ~60–100 μm) the tensile strength was measured as the load at which the sample failed. Before determining the mechanical characteristics, the films were prepressed for densification. The value of the breaking stress was increased with the increase in the mass fraction of F-2M. The maximum value of the breaking stress of a thin-film composite membrane was ~7 MPa for x = 0.3 (44 vol.%). The relatively high characteristic of the tensile strength of ~3.5 MPa for x = 0.25 (37.7 vol.%) was also shown (Figure 7). The obtained tensile strength values are high, close to [26], and suitable for intermediate fuel cells.

## 4. Conclusions

The proton conductivity of the polymer systems (1-x)CsH_2_PO_4_-xF-2M (x = 0–0.3) was first synthesized and analyzed, along with XRD and FTIR spectra. F-2M is shown to be the promising polymer for the formation of the membranes (1-x)CsH_2_PO_4_-xF-2M, which exhibits the high values of proton conductivity and mechanical strength. It has been shown that F-2M is a chemically inert matrix for CsH_2_PO_4_ polymer composites. The most optimal method for obtaining the thin non-porous films with the high conductivity was proposed. The use of a mixture of the solvents with different boiling points made it possible to obtain the dense thin-film (60–100 μm) membranes at high evaporation rates. The distribution of the particles in the polymer volume is close to the uniform. The FTIR spectra XRD data confirm the stability of the CsH_2_PO_4_ in the polymer composites with insignificant changes due to the interface interaction and the salt dispersion. Despite a decrease in the composite conductivity compared to the pure CsH_2_PO_4_ due to the polymer additive, the values in the high-temperature region remain high enough (10^−2^–3·× 10^−3^ S/cm) to enable the use of (1-x)CsH_2_PO_4_-xF-2M as proton membranes. The long-term stability of the proton membranes was shown. This creates the prospects for using polymer F-2M additives to synthesize proton membranes based on CsH_2_PO_4_ for medium-temperature fuel cells and other electrochemical devices. However, further improvements such as pretreatment of the salt suspension using a bead mill are needed to obtain the thinner gas-impermeable films with significant proton transport characteristics. 

## Data Availability

Not applicable.

## Supplementary Materials

The following supporting information can be downloaded at: https://www.mdpi.com/article/10.3390/membranes13050520/s1, Figure S1: The conductivity dependence as a function of the time holding at T = 245 °C.
