# Supplementary material for: Copolymer of VDF/TFE as a Promising Polymer Additive for CsH2PO4-Based Composite Electrolytes
Source: Membranes (Basel). 2023 May 17;13(5):520. doi: 10.3390/membranes13050520 (PMC10224548; doi:10.3390/membranes13050520)
Supplement: Supplementary file 1 [file membranes-13-00520-s001.zip › membranes-2385871-supplementary.pdf]

Copolymer of VDF/TFE as a promising polymer additive for CsH_2PO_4-based composite electrolytes

[1,2]Yuri Kungurtsev, [1,2]Irina Bagryantseva, [1]Valentina Ponomareva*

1 Institute of Solid State Chemistry and Mechanochemistry SB RAS, Russia, Novosibirsk
2 Novosibirsk State University, Russia, Novosibirsk
* Correspondence: ponomareva@solid.nsc.ru

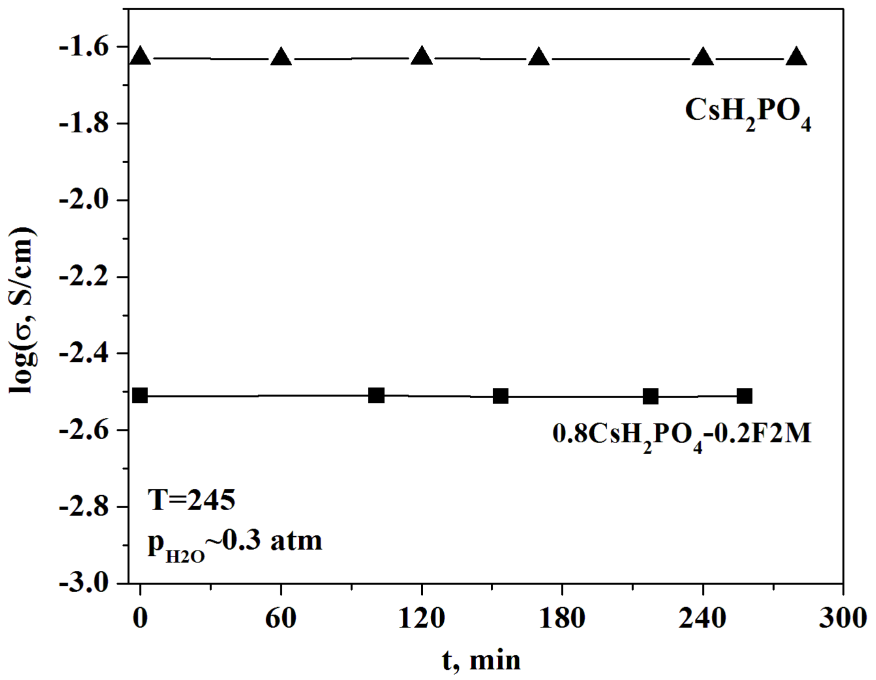

Figure S1. The conductivity dependence as a function of the time holding at T=245°C.

The storage of the sample x=0.2 at the high temperatures (T=245°C, pH_2O~0.3 atm) for ~4 hours shows the short term stability of the proton conductivity values for the membranes. The PXRD of the used samples also confirmed the immutability of the structural properties of CsH_2PO_4- F-2M polymer systems.
